# Response to: Comment on: “Is There Evidence for the Development of Sex-Specific Guidelines for Ultramarathon Coaches and Athletes? A Systematic Review”

**DOI:** 10.1186/s40798-023-00618-z

**Published:** 2023-09-08

**Authors:** Claudia P. M. G. Kelly

**Affiliations:** College of Medicine and Health, The University of Exeter, St Luke’s Campus, Heavitree Road, Exeter, EX1 2LU UK

Dear Editor,

I would like to begin by thanking da Silva and Benjamim [1] for their interest in my systematic review [2] on whether or not enough evidence exists to support the development of sex-specific guidelines for ultramarathon runners. In their letter to the editor [1], da Silva and Benjamim raise concerns about the validity of the conclusions of the review due to the methodology followed. They cover several points which will be responded to here.

Firstly, the letter [1] states that an incorrect reference was used when referring to the PRISMA guidelines. I acknowledge this error and thank da Silva and Benjamim for bringing this to my attention. However, I do not believe that this led to “mistakes in writing the review results”. Perhaps the correspondents could clarify what they mean by this.

Secondly, the correspondents [1] refer to my statement that “The methods were specified in advance and documented in a detailed protocol” and dispute this because the protocol was not registered in a database. This statement is in fact correct and the protocol is available for review via email correspondence. It is true that the protocol was not registered prior to the review being carried out; however, the free availability of the protocol means that the reproducibility of the review is not affected. I agree that registration of the protocol would have enhanced the credibility of the review. The reason the protocol was not registered was because the study was carried out as part of a postgraduate research project and, at the time the review commenced, publication was not considered. This is also the reason behind the sole authorship, which is acknowledged in the limitations section of the paper in question [2].

The letter [1] also posits that there was insufficient information included in the flowchart (Fig. 1) [2] with regards to the exclusion of studies. The correspondents state that “it is unclear to the reader why the author excluded such studies”. The terms ‘ineligible outcome’ and ‘ineligible population’ represent the fact that the studies in question did not meet the clearly stated eligibility criteria for inclusion in the systematic review. A study excluded due to an ‘ineligible population’ was excluded as the study participants did not meet the following criteria: “Male and female ultrarunners (defined as individuals who have completed a race > 42.2 km) aged 18 years and above, who compete or participate in single-day or multi-day events” [2]. For example, studies of runners < 18 years of age were excluded. It is my opinion that this is not ‘unclear’ and does not compromise the ‘transparency’ of the inclusion and exclusion of studies in the review [2].

**Fig. 1:**
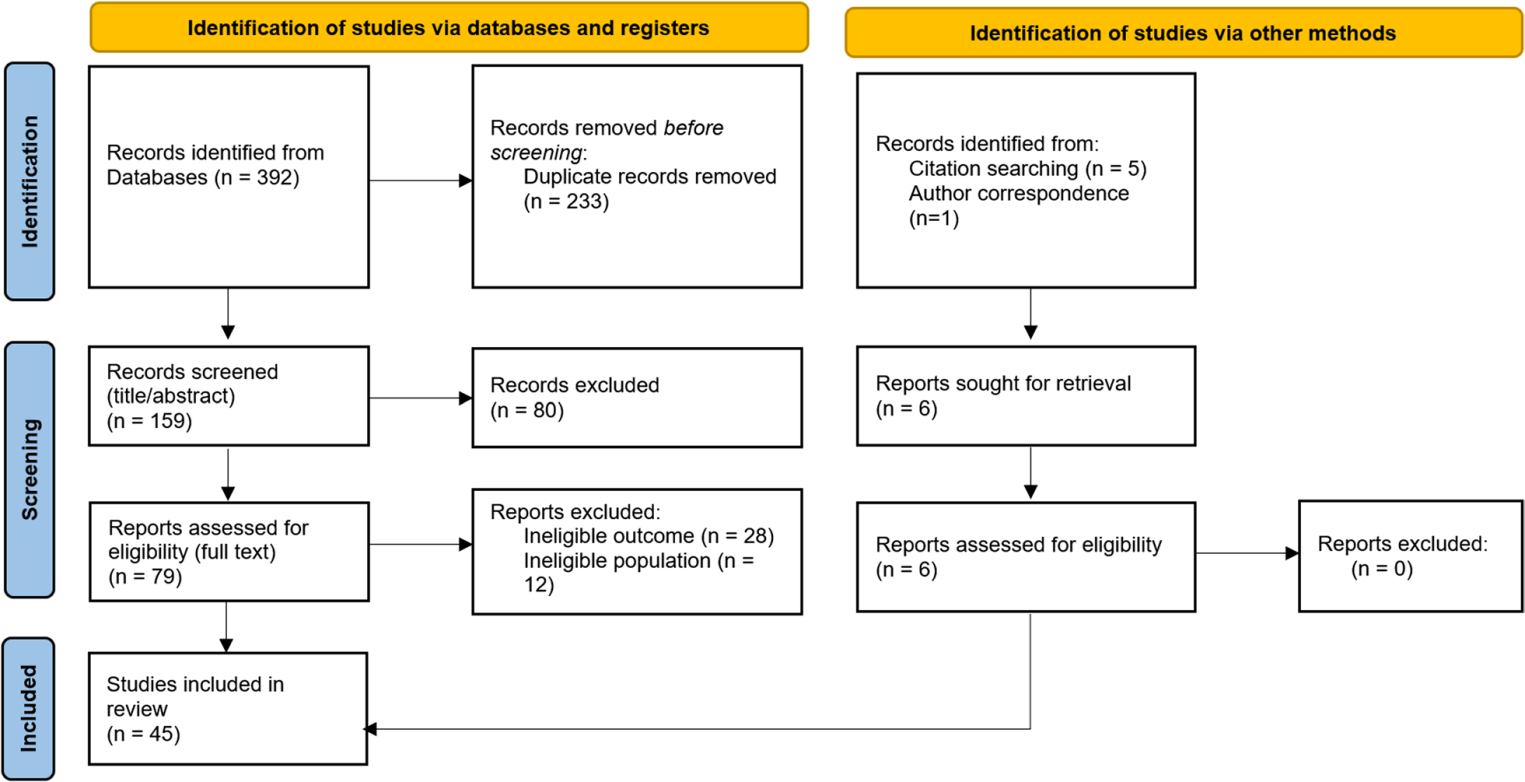
Summary of search strategy

With regards to the research question, the letter [1] states that the research question was based on the P-population/I-intervention/C-comparison/O-outcome (PICO) model and that this is inaccurate. Actually, it is the inclusion criteria which utilise the PICO model, not the research question. This outlines the PICO criteria of the studies which met the criteria to be included in the review [2]. The study designs included in the review were not erroneously described under the I-intervention label as the authors claim. It was merely noted that observational studies with no intervention were not excluded [2].

In the concluding remarks of my review [2], I state that “when considered as a whole, the body of research currently suggests that sex-specific recommendations and guidelines could improve performance and health outcomes in female ultramarathon runners”. The letter [1] implies that this statement is inappropriate given the overall poor quality of studies included in the review [2]. I disagree that this statement is unfounded. It is true that many studies have found differences between male and female ultrarunning athletes, and that while the overall quality of the literature is low, the pooled results do *suggest* that sex-specific guidelines *could* improve outcomes in female ultramarathon runners. Furthermore, the above statement was followed by the proviso that “the evidence base is currently insufficient to formulate such guidelines, and further research that recognises sex as an important bivariate measure is required”. I was careful not to make inappropriate recommendations or draw inferences based on the results of the studies included in the review on account of the preponderance of observational designs. I believe that my review [2] summarises the current body of research and emphasises the many areas of deficiency. Aside from addressing the question in the title, the review was intended to stimulate discussion and highlight the need for larger female cohorts and the inclusion of sex as a bivariate measure in future ultramarathon research. Only then will the development of scientifically robust recommendations for female ultrarunners be possible.

## References

[1] Lopes da Silva LS, Benjamim CJR. Comment on: “Is there evidence for the development of sex-specific guidelines for ultramarathon coaches and athletes? A systematic review.” Sports Med Open. 2023. 10.1186/s40798-023-00615-2 PMC10482810 37673811

[2] Kelly C (2023). Is there evidence for the development of sex-specific guidelines for ultramarathon coaches and athletes? A systematic review. Sports Med Open.

